# Incidence and prognosis of myocardial injury in patients with severe trauma

**DOI:** 10.1007/s00068-021-01846-2

**Published:** 2021-12-08

**Authors:** Alexandra Stroda, Simon Thelen, René M’Pembele, Antony Adelowo, Carina Jaekel, Erik Schiffner, Dan Bieler, Michael Bernhard, Ragnar Huhn, Giovanna Lurati Buse, Sebastian Roth

**Affiliations:** 1 Department of Anesthesiology, Medical Faculty and University Hospital Duesseldorf, Heinrich-Heine-University Duesseldorf, Moorenstr. 5, 40225 Duesseldorf, Germany; 2 Department of Orthopedics and Trauma Surgery, Medical Faculty and University Hospital Duesseldorf, Heinrich-Heine-University Duesseldorf, Moorenstr. 5, 40225 Duesseldorf, Germany; 3 Emergency Department, Medical Faculty and University Hospital Duesseldorf, Heinrich-Heine-University Duesseldorf, Moorenstr. 5, 40225 Duesseldorf, Germany

**Keywords:** Troponin, Cardiac biomarkers, Resuscitation room, Multiple trauma, Mortality

## Abstract

**Purpose:**

Severe trauma can lead to end organ damages of varying severity, including myocardial injury. In the non-cardiac surgery setting, there is extensive evidence that perioperative myocardial injury is associated with increased morbidity and mortality. The impact of myocardial injury on outcome after severe trauma has not been investigated adequately yet. We hypothesized that myocardial injury is associated with increased in-hospital mortality in patients with severe trauma.

**Materials/methods:**

This retrospective cohort study included patients ≥ 18 years with severe trauma [defined as injury severity score (ISS) ≥ 16] that were admitted to the resuscitation room of the Emergency Department of the University Hospital Duesseldorf, Germany, between 2016 and 2019. The main endpoint was in-hospital mortality. Main exposure was myocardial injury at arrival [defined as high-sensitive troponin T (hsTnT) > 14 ng/l]. For statistical analysis, receiver operating characteristic curve (ROC) and multivariate binary logistic regression were performed.

**Results:**

Out of 368 patients, 353 were included into statistical analysis (72.5% male, age: 55 ± 21, ISS: 28 ± 12). Overall in-hospital mortality was 26.1%. Myocardial injury at presentation was detected in 149 (42.2%) patients. In-hospital mortality of patients with and without myocardial injury at presentation was 45% versus 12.3%, respectively. The area under the curve (AUC) for hsTnT and mortality was 0.76 [95% confidence interval (CI) 0.71–0.82]. The adjusted odds ratio of myocardial injury for in-hospital mortality was 2.27 ([95%CI 1.16–4.45]; *p* = 0.017).

**Conclusion:**

Myocardial injury after severe trauma is common and independently associated with in-hospital mortality. Thus, hsTnT might serve as a new prognostic marker in this cohort.

**Supplementary Information:**

The online version contains supplementary material available at 10.1007/s00068-021-01846-2.

## Introduction

Severe trauma accounts for a great number of deaths worldwide and still is one of the leading causes of death among those under 40 years [1]. As the incidence of severe trauma is high, prediction of outcome is still a subject of current research. In this context, several prognostic scores, such as the revised trauma score (RTS) or the trauma and injury severity score (TRISS) have been suggested [2–5]. However, these scores do not take into account end organ damages apart from trauma-associated physical injuries which might also play a substantial role. One of these end organ damages in severely injured patients might be myocardial injury. According to the fourth universal definition of myocardial infarction, any detection of troponin exceeding the 99th percentile represents myocardial injury, independent of its pathophysiological mechanism [6]. In the non-cardiac surgery setting, there is extensive evidence that perioperative myocardial injury is associated with higher mortality and an increased rate of postoperative major adverse cardiovascular events (MACE) [7]. In addition to direct chest trauma, several potential other pathomechanisms postulated myocardial injury after surgery, i.e., hemodynamic disturbances due to acute bleeding, as well as activation of coagulation and inflammation. These mechanisms are also present in trauma [8–10]. As high-sensitivity troponin assays are able to detect troponin release less than 1 h after an initial event, measurement of troponin at presentation after trauma might detect both, direct myocardial injury by immediate chest trauma and secondary myocardial injury due to a mismatch of oxygen supply and demand. The impact of myocardial injury on outcome after severe trauma is underexplored. Therefore, we hypothesized that myocardial injury at hospital presentation regardless of its cause is associated with increased in-hospital mortality after severe trauma.

## Methods

The present study is a retrospective, single-center cohort study, approved by the ethical committee of the Heinrich-Heine-University Duesseldorf, Germany (Reference number 2020–1122). The study was conducted in accordance with the guidelines for good clinical practice (GCP) and the declaration of Helsinki. All handling of personal data complied with the GCP Guidelines and followed the General Data Protection Regulation (EU) 2016/679. This manuscript follows the STROBE reporting guidelines for retrospective cohort studies. The research question was formulated in compliance with the PICO format.

### Participants

Severely injured patients [defined as injury severity score (ISS) ≥ 16] and patients who were  ≥ 18 years of age admitted to the resuscitation room of the Emergency Department of the University Hospital Duesseldorf (Level I trauma center) between January 2016 and December 2019 were included in the study [11, 12]. Patients were excluded when no measurement of troponin at arrival in the resuscitation room was available. The treatment of all patients was standardized according to Advanced Trauma Life Support (ATLS) guidelines.

### Exposures and main endpoint

The main exposure was myocardial injury as defined by the fourth universal definition of myocardial infarction, detected using high-sensitive troponin T (hsTnT; Roche Diagnostics, Elecsys®)[6]. HsTnT was measured immediately after arrival in the resuscitation room (main exposure) as well as on day 1 (= 24 h after trauma) and day 2 (= 48 h after trauma) (secondary exposures). The main endpoint of this study was in-hospital mortality.

### Sample size

Due to the retrospective nature of the study, we did not conduct a formal sample size calculation. Between 2016 and 2019, on average approximately 100 patients/year met the inclusion criteria and were defined as having adult severe trauma (ISS ≥ 16). Based on the current literature, we expected an all-cause in-hospital mortality of approximately 25% (= 100 expected events in total) [13]. Assuming that hsTnT values would be available in > 90% of patients, a sample size of 360 patients was deemed appropriate to answer the research question including robust multivariable adjustment with nine co-variables according to Peduzzi et al. [14].

### Statistical analysis

A complete case analysis was conducted. Categorical data were presented as absolute numbers (percent). Continuous data were presented as mean ± standard deviation or median (interquartile range) as applicable. The discrimination of hsTnT for in-hospital mortality was examined by receiver operating characteristic curve (ROC) and the area under the curve (AUC). To quantify the association between myocardial injury and the main endpoint, multivariate binary logistic regression with forced entry of predefined co-variables was conducted. For continuous variables, the established cutoff (hsTnT) or data-driven cutoff (age, base excess) was used. The coding and definitions for each covariable can be found in supplementary Table S3. Hosmer–Lemeshow test was performed to evaluate goodness of fit. In addition, we evaluated the additional value of myocardial injury when added to the ISS score in a separate logistic regression model. We also calculated the AUCs for all logistic regression models with and without myocardial injury to determine potential improvement and performed De Long-Test for comparison of AUCs. During review process, a “post-factum” Kaplan–Meier analysis was performed.

### Predefined co-variables

The following co-variables for multivariable adjustment were predefined: age, sex, ISS, pre-existing chronic kidney injury [as defined by Kidney Disease Improving Global Outcome (KDIGO) Criteria], pre-existing coronary artery disease, base excess[15], American Society of Anesthesiologists (ASA) classification and thorax trauma (defined as any form of physical injury to the chest including the ribs, muscles, heart and lungs). The choice of co-variables was driven by its potential impact on both troponin and outcome and based on literature research by two independent members of the study team.

## Results

**Fig. 1:**
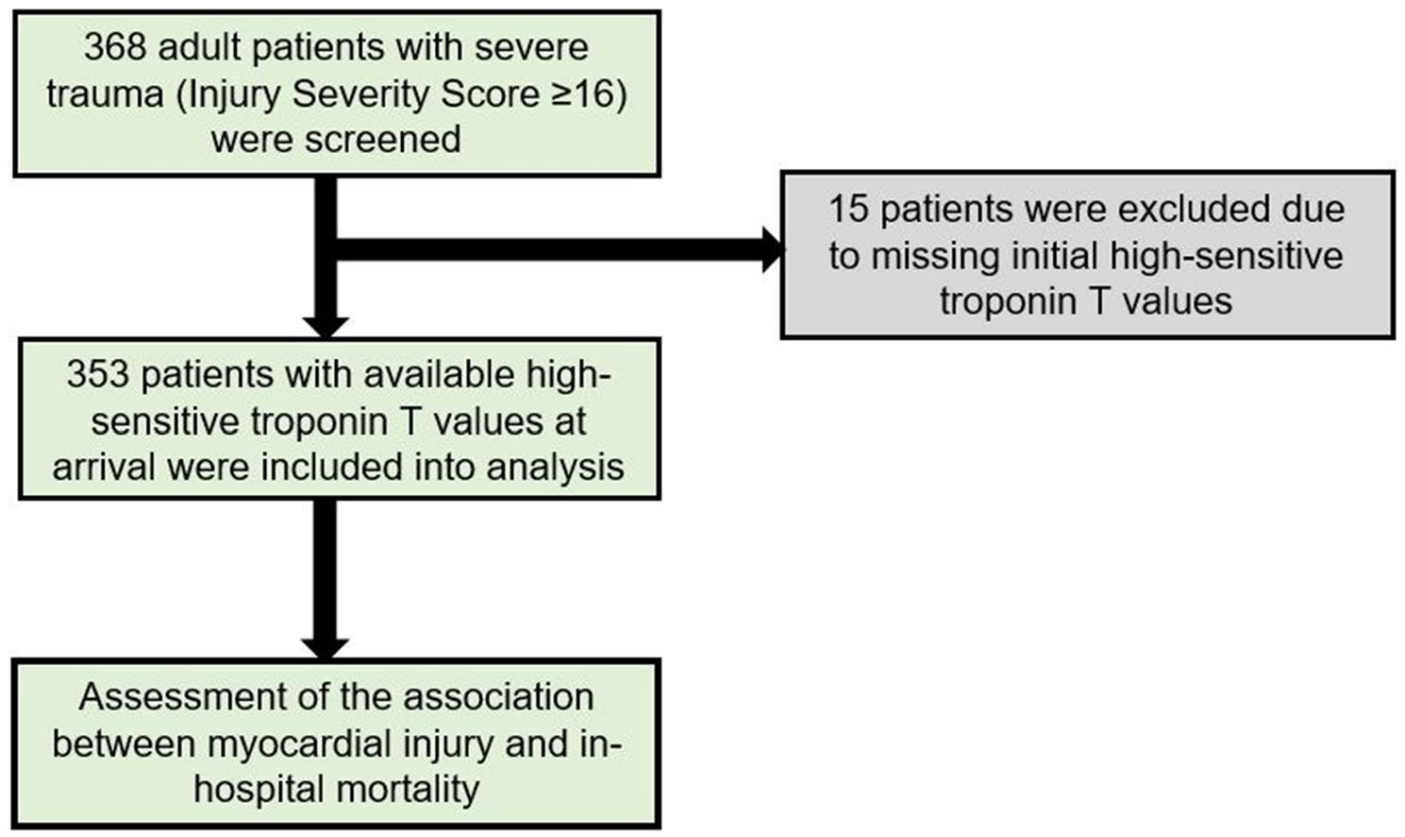
Study flowchart showing selection process of the study cohort

In total, 368 patients were screened for inclusion, thereof 15 patients had to be excluded due to missing hsTnT values at admission to the resuscitation room of the Emergency Department. Data on the main endpoint and all other co-variables were complete (see Fig. 1). Patient characteristics are presented in Table 1. Mean hsTnT at presentation was 63.33 ± 415.72. At arrival, 149/353 (42.2%) patients presented with myocardial injury (hsTnT > 14 ng/l). Overall in-hospital mortality was 26.1% (92 / 353 patients). Mortality was 45% (67 of 149 patients) versus 12.3% (25 of 204 patients) among patients with and without myocardial injury at presentation, respectively. 179/353 (50.7%) patients had thorax trauma. In patients with myocardial injury, thorax trauma was present in 81/149 (54.4%) patients. Due to missing values or death, 108/353 (30.6%) patients had hsTnT measurement on day 1 and 110/353 (31.2%) patients on day 2.

**Table 1:** Patient characteristics

	Patients with severe trauma (*n* = 353)	Patients with initial myocardial injury (*n* = 149)	Patients without initial myocardial injury (*n* = 204)
*Baseline characteristics*			
Male sex no. (%)	256 (72.5%)	104 (69.8%)	152 (74.5%)
Age (years)	55 ± 21	62 ± 22	50 ± 18
*Comorbidities*			
Coronary artery disease	28 (7.9%)	16 (10.7%)	12 (5.9%)
Chronic kidney disease (≥ CKD III)	9 (2.5%)	7 (4.7%)	2 (1.0%)
Diabetes mellitus	22 (6.2%)	9 (6.0%)	13 (6.4%)
Arterial hypertension	85 (24.1%)	41 (27.5%)	44 (21.6%)
ASA physical status			
* ASA I*	159 (45%)	47 (31.5%)	112 (54.9%)
* ASA II*	111 (31.4%)	57 (38.3%)	54 (26.5%)
* ASA III*	52 (14.7%)	24 (16.1%)	28 (13.7%)
* ASA IV*	7 (2.0%)	5 (3.4%)	2 (1.0%)
*Trauma related data*			
ISS	28 ± 12	31 ± 12	26 ± 10
GCS at arrival	3 [3–14]	3 [3–9]	10 [3–15]
Thorax trauma	179 (50.7%)	81 (54.4%)	98 (48.0%)
*Laboratory values*			
Hb (mg/dl)	12.3 ± 2.4	11.6 ± 2.5	12.8 ± 2.1
INR	1.4 ± 0.8	1.6 ± 1.0	1.2 ± 0.5
PTT (sec)	31.75 ± 24.4	38.0 ± 32.6	27.3 ± 15.0
Base excess	– 3.8 ± 5.7	– 5.6 ± 6.7	– 2.5 ± 4.5
HsTnT initial (ng/ml)	63.33 ± 415.72	139.8 ± 633.1	7.46 ± 3.0
Creatinine initial (mg/dl)	1.04 ± 0.62	1.16 ± 0.51	0.95 ± 0.68
HsTnT 24 h (ng/ml)	134.80 ± 271.28	199.40 ± 332.06	44.36 ± 96.16
HsTnt 48 h (ng/ml)	477.75 ± 2900.88	865.75 ± 4004.0	60.45 ± 177.85
*Outcome*			
Death in hospital	92 (26.1%)	67 (45%)	25 (12.3%)

Values are presented as *N* (%) or mean (± SD)/median (IQL), where appropriate *ASA *American Society of Anesthesiologists, *ISS *injury severity score, *gCS *Glasgow Coma Scale, *Hb *hemoglobin, *INR *international normalized ratio, *PTT *partial thromboplastin time, *HsTnT *high-sensitive troponin

### ROC analysis

**Fig. 2:**
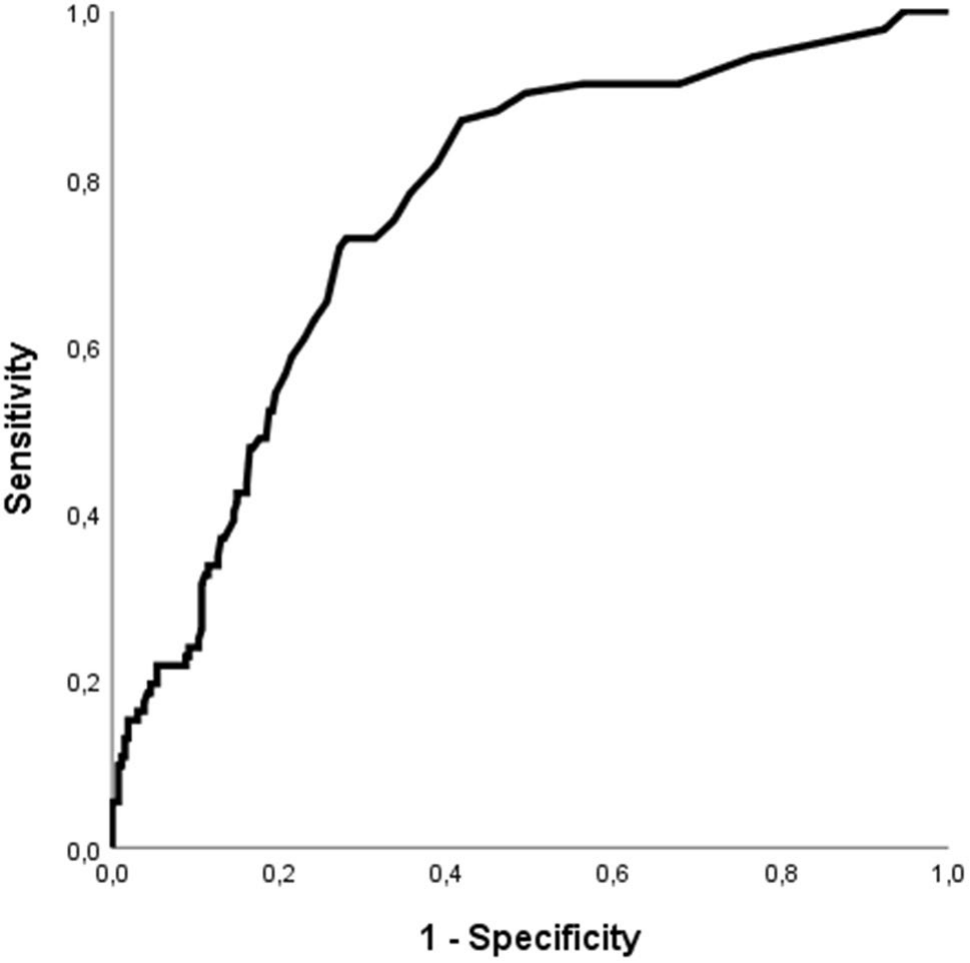
Receiver operating characteristics (ROC) curve showing the discrimination of initial high-sensitive troponin T (hsTnT) for in-hospital mortality. ROC analysis revealed an AUC of 0.76 [95% confidence interval (CI) 0.71–0.82]

**Fig. 3:**
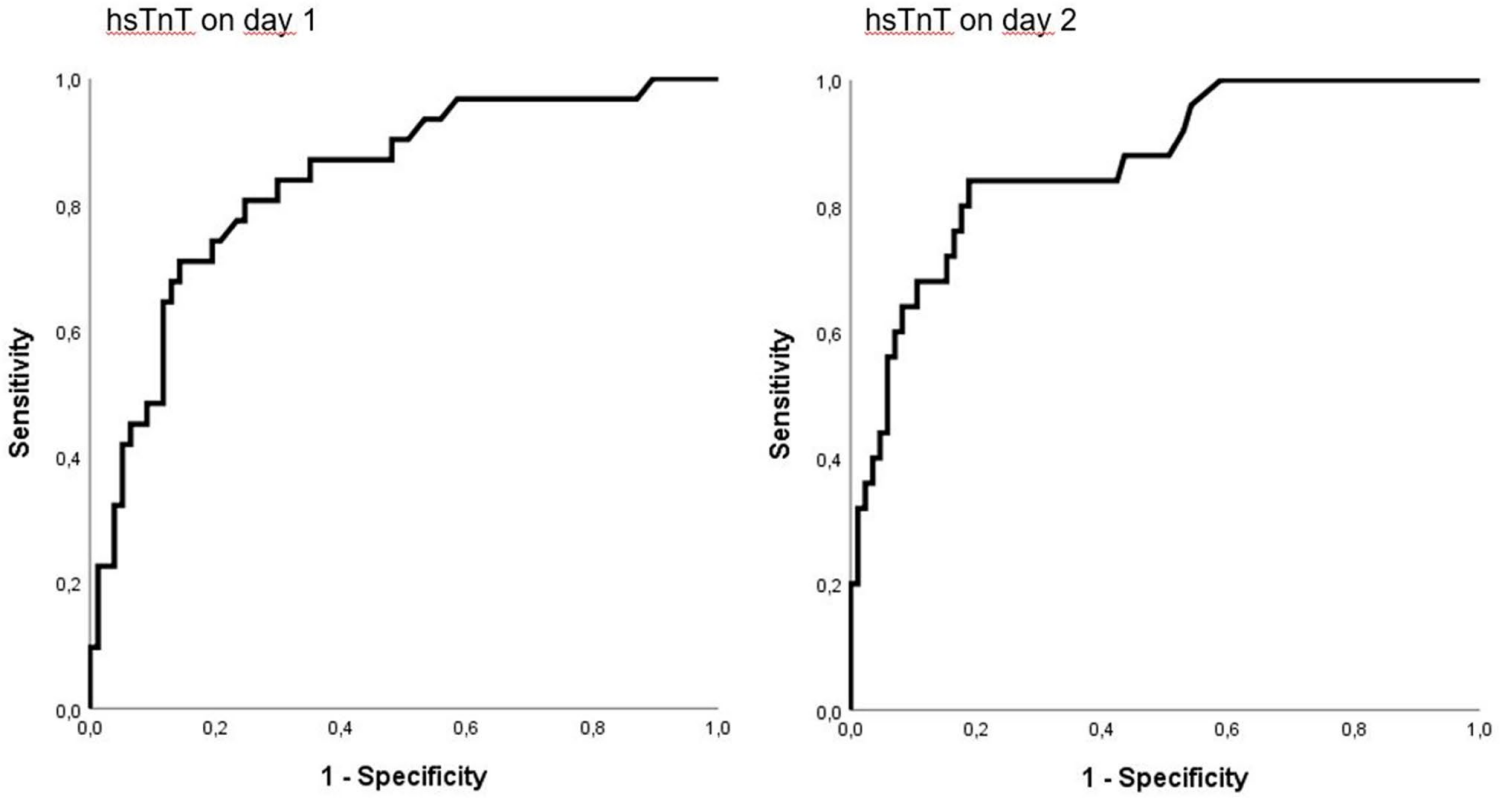
Receiver operating characteristics (ROC) curves showing the discrimination of high-sensitive troponin T (hsTnT) on day 1 and 2 for in-hospital mortality. ROC analysis of hsTnT on day 1 revealed an AUC of 0.84 [95% CI 0.75–0.92]. The AUC of hsTnT on day 2 was 0.87 [95%CI 0.79–0.95]

The AUC of initial hsTnT for in-hospital mortality was 0.76 [95% confidence interval (CI) 0.71–0.82] (see Fig. 2). The AUC for hsTnT and in-hospital mortality on day 1 and 2 was 0.84 [95% CI 0.75–0.92] and 0.87 [95%CI 0.79–0.95], respectively (see Fig. 3).

### Multivariate binary logistic regression

Multivariate binary logistic regression revealed an adjusted odds ratio (OR) of 2.27 [95% CI 1.16–4.45] for myocardial injury and in-hospital mortality (see Table 2). Hosmer–Lemeshow test revealed a *p* value of 0.3. The full results of multivariate analysis are reported in Table 2. The results of univariate analysis for each covariable are presented in supplementary Table S1.

**Table 2:** Multivariate binary logistic regression model

Variable	Regression coefficient	Odds ratio	95% Confidence interval		*p* value
			Lower	Upper	
Age	1.611	5.01	2.35	10.70	** < 0.001**
Myocardial Injury	0.82	2.27	1.16	4.45	**0.017**
ISS Score	0.052	1.05	1.02	1.08	** < 0.001**
Sex	– 0.07	0.93	0.45	1.90	0.833
ASA physical status	– 0.10	0.91	0.58	1.41	0.671
Thorax trauma	– 0.69	0.50	0.25	1.00	0.052
Chronic kidney disease	– 0.96	0.38	0.06	2.39	0.304
Coronoary artery disease	0.84	2.35	0.80	6.66	0.123
Base excess	1.78	5.63	2.83	11.20	** < 0.001**
Constant term	– 4.3	0.01			** < 0.001**

Significant results are presented as bold *p*-values

*ISS *injury severity score, *ASA *American Society of Anesthesiologists

### AUC of logistic regression models

The AUC for the above described logistic regression model was 0.85 [95% CI 0.80–0.90] without myocardial injury and 0.86 [95% CI 0.81–0.90] including myocardial injury. De Long test showed that the difference between the AUCs was not significant.

### Additional value of myocardial injury when added to ISS Score

The AUC of a univariate logistic regression model including ISS and in-hospital mortality was 0.67 [95% CI 0.60–0.73]. The AUC improved to 0.76 [95% CI 0.71–0.82] when adding myocardial injury to this model. According to De Long Test, this improvement was significant with a difference between AUCs of 0.09 ([95% CI 0.04  – 0.15]; *p* = 0.0007).

### Survival analysis

Kaplan–Meier analysis revealed that in-hospital survival rates of patients with and without myocardial injury are significantly different (*p* < 0.001, see supplementary Figure S1).

## Discussion

The results of this study demonstrate that myocardial injury at arrival in the resuscitation room is common and independently associated with in-hospital mortality among severely injured patients. Furthermore, the addition of myocardial injury to the established ISS score was able to improve prognostic value significantly.

### Troponin as a predictor of mortality in severe trauma patients in the literature

Varying pathomechanisms leading to myocardial injury in severe trauma patients are discussed [9, 10, 16]. To date, only few studies investigated this aspect in the past [17–19]. Keskpaik et al. retrospectively investigated 147 severely injured patients with chest trauma [defined as Chest Abbreviated Injury Scale (AIS) > 3][17]. The primary endpoints of this study were in-hospital mortality and 1-year mortality. The authors found that elevated troponin is associated with poor outcome compared to patients with normal troponin levels. As only patients with chest trauma were included, a selection bias might be present in this study not considering alternative pathomechanisms such as a mismatch between oxygen supply and demand. Edouard et al. postulated in another analysis including 728 patients that elevated troponin I is not associated with higher mortality in trauma patients [20]. However, the authors concluded that troponin I assessment may be used for initial screening in high-risk trauma patients to detect anatomical cardiac injuries. While interpreting the findings listed above, it has to be taken into account that definition of trauma and measurement of troponin (troponin I versus troponin T) differ between the examined cohorts. Moreover, Edouard et al. did not give clear definitions of “late mortality” and trauma severity and did only distinguish between troponin release yes/no rather than considering troponin as continuous variable. Mahmood et al. also identified troponin as a risk marker for mortality in severe trauma patients, but did predominantly concentrate on patients with chest trauma, in whom traumatic damage of the heart plays a crucial role [17, 21]. The authors performed a retrospective analysis of 993 blunt traumatic chest injury patients divided into two groups (positive vs. negative troponin T) and concluded that patients with elevated troponin tend to have the worst outcome even in the absence of clinical evidence of acute cardiac involvement.

The findings of our study add to the limited literature in this field. We investigated a broad and representative cohort of patients with severe trauma. Especially, we included all types of injuries taking into account other mechanisms of troponin release than chest trauma, and according to our multivariate analysis, the association between myocardial injury and mortality was independent of chest trauma. Although we performed a retrospective analysis, our data are based on a prospectively constituted database which ensures high data quality. In addition, we did not only investigate initial troponin, but also included measurements on day 1 and 2 after trauma which enables the interpretation of troponin values over time. Finally, we evaluated the additional value of myocardial injury when added to the established ISS and our results show clearly that the combination of myocardial injury and ISS is significantly better in terms of prognosis than ISS alone.

### Strengths and limitations

Our study has several strengths and limitations. Strengths: Because of the standardized collection of data in patients enrolled in the TraumaRegistry®, data quality of this study can be regarded as high. Moreover, we conducted robust multivariate regression including nine relevant covariates and the choice of these covariates was based on literature research. Limitations: One limitation of this study consists of the retrospective single-center design. However, our results for the main endpoint in-hospital mortality are in line with the current literature and thus can be considered as representative for our study cohort. A second limitation refers to ROC analysis for troponin and mortality on day 1 and 2. It is important to clarify that mainly patients with elevated initial troponin at the resuscitation room might have received further troponin sampling which surely led to relevant selection bias. Third, this study only investigated the association between myocardial injury and in-hospital mortality. Future studies should investigate the association with further relevant endpoints (e.g. in-hospital complications, hospital length of stay or long-term outcome).

## Conclusions

Myocardial injury after severe trauma is common and independently associated with in-hospital mortality. Thus, hsTnT may serve as a new prognostic marker in this cohort. These results are clinically relevant and troponin may be considered to be included into established risk scores for trauma patients.

## Supplementary Information

Below is the link to the electronic supplementary material.

Supplementary file1 (PDF 94 KB)

Supplementary file2 (DOCX 157 KB)

## Data Availability

The datasets generated during and/or analyzed during the current study are available from the first author on reasonable request.
