# Supplementary figures and images for: Incidence and prognosis of myocardial injury in patients with severe trauma
Source: Eur J Trauma Emerg Surg. 2021 Dec 8;48(4):3073–9. doi: 10.1007/s00068-021-01846-2 (PMC9360164; doi:10.1007/s00068-021-01846-2)

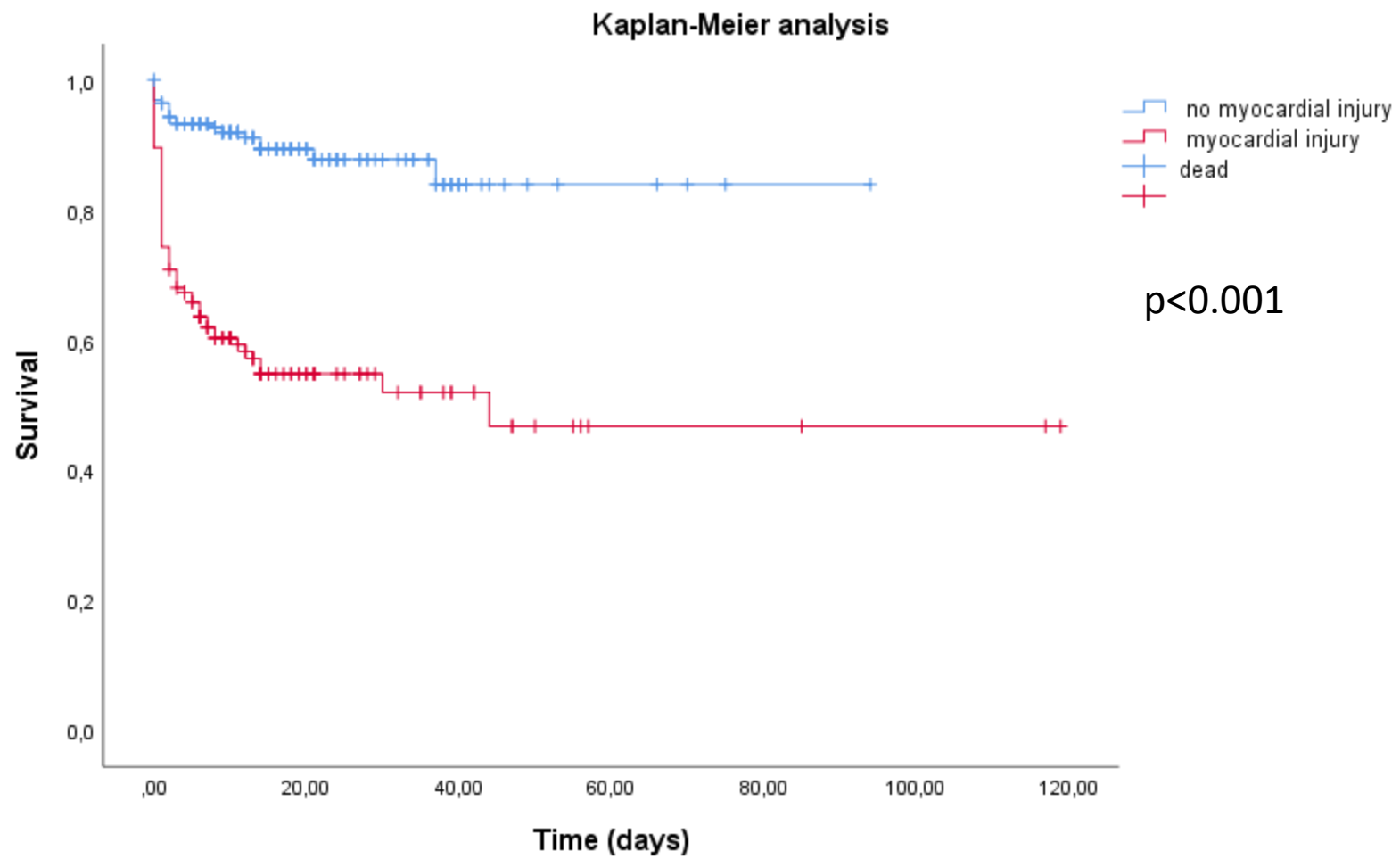

Supplement: Supplementary file 1 — Supplementary file1 (PDF 94 KB) [file 68_2021_1846_MOESM1_ESM.pdf]
