# Supplementary material for: Incidence and prognosis of myocardial injury in patients with severe trauma
Source: Eur J Trauma Emerg Surg. 2021 Dec 8;48(4):3073–9. doi: 10.1007/s00068-021-01846-2 (PMC9360164; doi:10.1007/s00068-021-01846-2)
Supplement: Supplementary file 2 — Supplementary file2 (DOCX 157 KB) [file 68_2021_1846_MOESM2_ESM.docx]

**Table S1: Univariate analysis for predefined covariables included into multivariate logistic regression**

| **Variable** | **Regression Coefficient** | **Odds Ratio** | **95% Confidence interval** | | **p-value** |
| --- | --- | --- | --- | --- | --- |
|  |  |  | **lower** | **upper** |  |
| Age | 1.78 | 5.93 | 3.53 | 9.95 | **<0.001** |
| Myocardial Injury | 1.77 | 5.85 | 3.45 | 9.92 | **<0.001** |
| ISS-Score | 0.05 | 1.05 | 1.03 | 1.07 | **<0.001** |
| Sex | 0.20 | 1.22 | 0.72 | 2.05 | 0.460 |
| ASA physical status | 0.36 | 1.44 | 1.06 | 1.96 | **0.022** |
| Thorax trauma | -0.43 | 0.65 | 0.40 | 1.05 | 0.077 |
| Chronic kidney disease | 0.36 | 1.43 | 0.35 | 5.85 | 0.616 |
| Coronoary artery disease | 0.83 | 2.30 | 1.04 | 5.06 | 0.039 |
| Base excess | 1.58 | 4.85 | 2.89 | 8.16 | **<0.001** |

ISS=Injury Severity Score; ASA=American Society of Anaesthesiologists;

**Table S2: Post factum binary multivariate logistic regression model**

| **Variable** | **Regression Coefficient** | **Odds Ratio** | **95% Confidence interval** | | **p-value** |
| --- | --- | --- | --- | --- | --- |
|  |  |  | **lower** | **upper** |  |
| Age | 1.67 | 5.23 | 2.426 | 11.26 | **<0.001** |
| Myocardial Injury | 0.72 | 2.06 | 1.04 | 4.07 | **0.039** |
| ISS-Score | 0.05 | 1.05 | 1.02 | 1.08 | **0.003** |
| Sex | -0.04 | 0.96 | 0.46 | 1.98 | 0.905 |
| ASA physical status | -0.07 | 0.93 | 0.60 | 1.45 | 0.75 |
| Thorax trauma | -0.66 | 0.52 | 0.26 | 1.05 | 0.066 |
| Chronic kidney disease | -0.48 | 0.62 | 0.09 | 4.28 | 0.627 |
| Coronoary artery disease | 0.841 | 2.32 | 0.80 | 6.75 | 0.123 |
| Base excess | 1.67 | 5.33 | 2.67 | 10.64 | **<0.001** |
| Hemorrhagic shock | 0.02 | 1.02 | 0.99 | 1.038 | 0.211 |

ISS=Injury Severity Score; ASA=American Society of Anaesthesiologists;

**Table S3: Coding and definition of variables for multivariate logistic regression**

| **Variable** | **Coding** | **Definition** |
| --- | --- | --- |
| Age | > 63 / ≤ 63 years | Data-driven cutoff (Youden-index) |
| Myocardial Injury | yes / no | hsTnT >14ng/l / hsTnT ≤14ng/l |
| ISS-Score | continous | Inclusion only when ISS ≥16 |
| Sex | Male / female |  |
| ASA physical status | ASA class 1 /2 / 3 / 4 |  |
| Thorax trauma | yes / no | any physical injury to the chest |
| Chronic kidney disease | yes / no | CKD stadium ≥ 3 / CKD stadium < 3 |
| Coronoary artery disease | yes / no | Diagnosed via coronary angiography |
| Base excess | < -3.95 / ≥ -3.95 | Data-driven cutoff (Youden-index) |

ISS = Injury severity score; ASA = Amercian Society of Anesthesiologists; hsTnT = high-sensitive troponin T; CKD = Chronic Kidney Disease

**Supplementary figure S1:** Kaplan-Meier analysis revealed that in-hospital survival rates of patients with and without myocardial injury are significantly different (p<0.001).
